# Proangiogenic functions of an RGD-SLAY-containing osteopontin icosamer peptide in HUVECs and in the postischemic brain

**DOI:** 10.1038/emm.2017.241

**Published:** 2018-01-19

**Authors:** Hahnbie Lee, Yin-Chuan Jin, Seung-Woo Kim, Il-Doo Kim, Hye-Kyung Lee, Ja-Kyeong Lee

**Affiliations:** 1 Department of Anatomy, Inha University School of Medicine, Inchon, Republic of Korea; 2 Medical Research Center, Inha University School of Medicine, Inchon, Republic of Korea; 3 Department of Histology and Embryology, Binzhou Medical University, Yantai, China

## Abstract

Osteopontin (OPN) is a phosphorylated glycoprotein secreted into body fluids by various cell types. OPN contains arginine-glycine-aspartate (RGD) and serine-leucine-alanine-tyrosine (SLAY) motifs that bind to several integrins and mediate a wide range of cellular processes. In the present study, the proangiogenic effects of a 20-amino-acid OPN peptide (OPNpt20) containing RGD and SLAY motifs were examined in human umbilical vein endothelial cells (HUVECs) and in a rat focal cerebral ischemia model. OPNpt20 exerted robust proangiogenic effects in HUVECs by promoting proliferation, migration and tube formation. These effects were significantly reduced in OPNpt20-RAA (RGD->RAA)-treated cells, but only slightly reduced in OPNpt20-SLAA (SLAY->SLAA)-treated cells. Interestingly, a mutant peptide without both motifs failed to induce these proangiogenic processes, indicating that the RGD motif is crucial and that SLAY also has a role. In OPNpt20-treated HUVEC cultures, AKT and ERK signaling pathways were activated, but activation of these pathways and tube formation were suppressed by anti-α_v_β_3_ antibody, indicating that OPNpt20 stimulates angiogenesis via the α_v_β_3_-integrin/AKT and ERK pathways. The proangiogenic function of OPNpt20 was further confirmed in a rat middle cerebral artery occlusion model. Total vessel length and vessel densities were markedly greater in OPNpt20-treated ischemic brains, accompanied by induction of proangiogenic markers. Together, these results demonstrate that the 20-amino-acid OPN peptide containing RGD and SLAY motifs exerts proangiogenic effects, wherein both motifs have important roles, and these effects appear to contribute to the neuroprotective effects of this peptide in the postischemic brain.

## Introduction

Osteopontin (OPN) is a secretory phosphoprotein expressed in various tissues, including the brain, and it has been reported that OPN has important roles in various physiological processes, such as anchoring osteoclasts to the mineral bone matrix during bone resorption^1^ and attaching calcium oxalate crystals to renal tubules in stone formation.^2^ Elevation of OPN expression has been observed in various pathological conditions, including atherosclerosis,^3^ multiple sclerosis,^4^ rheumatoid arthritis^5^ and most cancers,^6^ which suggests that OPN has an important role in these conditions. In the normal brain, OPN expression is weak, but under pathological conditions, it is markedly upregulated in microglia and astrocytes.^7^ In particular, delayed but significant induction of OPN has been reported in ischemic stroke; for example, in a rat model of transient forebrain ischemia and in a mouse model of permanent focal ischemia, OPN induction began at 12 h after injury and peaked at 5 days, respectively.^8, 9^

OPN contains a highly conserved arginine-glycine-aspartic acid (RGD) motif in its N-terminal region, which interacts with α_v_β_1_-, α_v_β_3_- and α_v_β_5_-integrin.^10^ In addition, the SVVYGLR motif, which is adjacent to the RDG motif, is exposed by thrombin cleavage and binds to α_9_β_1_-, α_4_β_1_-, α_4_β_7_- and α_v_β_3_-integrin.^11, 12^ These OPN–integrin interactions mediate cell–cell and cell–matrix interactions and modulates a wide range of cellular processes, including adhesion, migration and survival,^13^ and have therefore been implicated in various diseases. In addition to integrins, OPN also binds to CD44 hyaluronate receptor, a transmembrane glycoprotein known to mediate inflammatory processes, cell adhesion, and cell migration^14^ via its CD44-binding domain located in the C-terminal region.^15^

In our previous study, we reported that an intranasally delivered RGD and serine-leucine-alanine-tyrosine (SLAY) motif-containing OPN icosamer peptide (20-amino-acid OPN peptide (OPNpt20)) exerted a robust neuroprotective effect in the postischemic brain because of its anti-inflammatory effects.^16^ In this previous study, OPNpt20 was found to significantly suppress inducible nitric oxide synthase (iNOS) induction and nitrite production in a rat model of focal cerebral ischemia (middle cerebral artery occlusion, MCAO) and in lipopolysaccharide-treated primary microglial cultures, and the results obtained suggested that the interaction between the RGD motif of OPNpt20 and α_v_β_3_-integrin has a critical role. In the present study, we investigated the proangiogenic potential of OPNpt20 in human umbilical vein endothelial cells (HUVECs) and in a rat MCAO model. In addition, we explored the molecular mechanism behind the proangiogenic effects of OPNpt20 using three mutant peptides (RGD→RAA, SLAY→SLAA and RGDSLAY→RAASLAA) and an α_v_β_3_-integrin-blocking antibody.

## Materials and methods

### Surgical procedure used for MCAO

Male Sprague–Dawley rats were housed under diurnal lighting conditions and allowed food and tap water *ad libitum*. All animal studies were carried out in strict accordance with the recommendations made in the Guide for the Care and Use of Laboratory Animals published by the National Institute of Health (2013) and ARRIVE guidelines (http://www.nc3rs.org/ARRIVE). The animal protocol used was reviewed and approved by the INHA University-Institutional Animal Care and Use Committee (INHA-IACUC; Approval Number INHA-160824-434). MCAO was carried out as described previously.^17^ In brief, 8-week-old male Sprague–Dawley rats (250–300 g) were anesthetized with 5% isoflurane in 30% oxygen/70% nitrous oxide and maintained using 0.5% isoflurane in the same gas mixture during surgery. Occlusion of the right middle carotid artery was induced for 1 h by advancing a nylon suture (4-0; AILEE, Busan, Korea) with a heat-induced bulb at its tip (~0.3 mm in diameter) along the internal carotid artery for 20–22 mm from its bifurcation with the external carotid artery. This was then followed by reperfusion for up to 7 days. A thermoregulated heating pad and a heating lamp were used to maintain a rectal temperature of 37±0.5 °C during surgery. Animals were randomly allocated to sham, MCAO+phosphate-buffered saline (PBS), MCAO+OPNpt20 or MCAO+OPNpt20-Db (mutant OPN peptide with both RGD and SLAY replaced) groups. Animals allocated to the sham group underwent an identical procedure, but the MCA was not occluded.

### Peptide treatment

**Figure 1:**
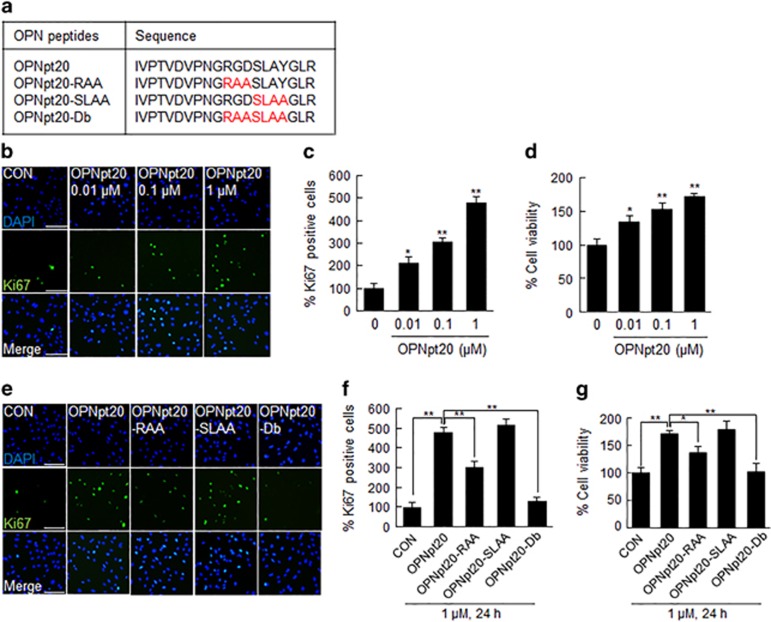
Induction of cell proliferation by 20-amino-acid OPN peptide (OPNpt20) and its mutants in human umbilical vein endothelial cell (HUVEC) cultures. (**a**) Amino-acid sequences of OPNpt20 and its three mutants. (**b**, **c**) Cell proliferation was visualized by immunofluorescent staining using anti-Ki67 antibody in HUVECs treated with OPNpt20 (0.01, 0.1 or 1 μM) for 24 h. Ki67 indices were determined by counting numbers of Ki67-positive cells among DAPI (4',6-diamidino-2-phenylindole)-positive cells in five high-power fields per plate in three plates. (**e**, **f**) HUVEC proliferation was visualized by immunostaining with anti-Ki67 antibody after treating HUVECs for 24 h with either of the three mutant peptides (1 μM). Representative images are presented (**b**, **e**) and results are presented as the mean±s.e.m. (*n*=3) (**c**, **f**). (**d**, **g**) MTT (3-(4,5-dimethylthiazol-2-yl)-2,5-diphenyl tetrazolium bromide) assays were conducted under the same conditions used in (**b**, **e**), and results are presented as the mean±s.e.m. (*n*=4). Scale bars, 200 μm. *P<0.05, **P<0.01 between the indicated groups.

OPNpt20 and three mutant peptides (Figure 1a) were custom-synthesized by Peptron (Daejeon, South Korea). Rats were anesthetized with an intramuscular injection of a ketamine (3.75 mg/100 g body weight) and xylazine hydrochloride (0.5 mg/100 g body weight) mixture. Peptides (1.7 μg/100 g body weight) were dissolved in 30 μl of PBS (0.01 M) and injected intranasally at 4, 5 and 6 days after MCAO. Brain tissues, cerebrospinal fluid and blood samples were collected at 7 days after MCAO.

### HUVEC cultures

HUVECs were purchased from the American Type Culture Collection (ATCC, Manassas, VA, USA). Cells were grown on 100 × 20 mm^2^ Petri dishes coated with 0.1% gelatin for subculture. Cultures were maintained with endothelial cell medium (Sciencell, Carlsbad, CA, USA) containing 5% fetal bovine serum (FBS), 1% penicillin/streptomycin and 1% endothelial cell growth supplement. Before peptide treatment, HUVEC cultures were starved in medium 199 (M199; Welgene, Gyeongsan, Korea) containing 20% endothelial cell medium for 12 h. Experiments were conducted using cells passaged three to eight times.

### MTT cell proliferation assay

HUVEC proliferation and viability were analyzed using an MTT (3-(4,5-dimethylthiazol-2-yl)-2,5-diphenyl tetrazolium bromide) assay. Briefly, HUVEC cultures were treated with peptide for 24 h; then, 10 μl of an MTT stock solution (5 mg ml^−1^ in 0.01 M PBS) was added and incubated for 1 h at 37 °C. Media were removed, and dimethyl sulfoxide (200 μl/24-well) was added to solubilize the formazan product. After 30 min at room temperature, the absorbance was measured at 460 nm.

### Immunocytochemistry

A total of 1.0 × 10^5^ cells per well were seeded on gelatin-coated cover glasses in 24-well plates. After starvation for 12 h, cultures were treated with peptide in M199 medium containing 1% FBS (GE Healthcare Life Sciences, Marlborough, MA, USA) for 24 h and then fixed with 4% paraformaldehyde. After washing with 0.01 M PBS, cells were incubated in blocking solution (0.1% Triton X-100, 2% bovine serum albumin, 5% FBS, 5% Normal goat serum in 0.01 M PBS) at room temperature for 1 h, immunostained with anti-Ki67 antibody (Abcam, Cambridge, UK) and mounted using a mounting medium with DAPI (4',6-diamidino-2-phenylindole; Vector Laboratories, Burlingame, CA, USA).

### Wound healing assay

HUVECs were seeded at 2.0 × 10^5^/well onto gelatin-coated 12-well plates and starved for 12 h after reaching 90% confluency. Wells were then scratched longitudinally and horizontally with a yellow tip. After washing two times with M199, cultures were treated with the same medium containing 1% FBS with or without OPNpt20. Cell migration was assayed 12 h after M199 treatment using a real-time cell history recorder (JuLi Stage; NanoEnTek, Seoul, South Korea). Wound widths were measured by using the ImageJ software MRI Wound Healing Tool (National Institute of Health (NIH), Bethesda, MD, USA), and percent cell motility was calculated using the following equation: ((area at 0 h−area at 12 h)/area at 0 h) × 100.

### Tube formation assay

HUVECs (5.0 × 10^4^) were seeded on Matrigel-coated wells of a 96-well plate with or without peptides for 12 h. Tube formation was quantified by measuring tube numbers and total tube lengths in four random × 3 magnification fields per well using a real-time cell history recorder (JuLi stage; NanoEnTek, Seoul, South Korea). The data analysis was performed using the Angiogenesis Analyzer in ImageJ (NIH).

### Immunoblot analysis

Brain homogenates or whole-cell lysates were extracted with RIPA buffer (50 mM Tris-HCl (pH 7.4), 150 mM NaCl, 1 mM EDTA, 0.5% NP40, 0.25% sodium deoxycholate, 0.5% Triton X-100, 10% glycerol and Complete Mini Protease Inhibitor Cocktail tablet (Roche Diagnostics, Basel, Switzerland)), and protein concentrations in extracts were determined using a bicinchoninic acid assay. Cell or tissue extracts were then loaded into 8–10% SDS-PAGE gels and immunoblotted using the following primary antibodies: anti-vascular endothelial growth factor (VEGF) (1:3000; Abcam, Cambridge, UK), anti-α-smooth muscle actin (1:500; Santa Cruz Biotechnology, Dallas, TX, USA), anti-matrix metallopeptidase 9 (MMP9) (1:3000, GeneTex, Irvine, CA, USA), anti-endothelial NOS (eNOS) (1:3000; Santa Cruz Biotechnology), anti-phospho-eNOS (1:3000; Cell Signaling Technology, Danvers, MA, USA), anti-neuronal NOS (nNOS) (1:3000; Santa Cruz Biotechnology), anti-phospho-nNOS (1:3000; Cell Signaling Technology), anti-integrin α_4_, anti-integrin α_9_, anti-integrin α_v_, anti-integrin-β_3_ (1:2000; Santa Cruz Biotechnology), anti-phosphatidylinositol-4,5-bisphosphate 3-kinase (PI3K), anti-phospho-PI3K, anti-ERK, anti-phospho-ERK, anti-Akt, anti-phospho-Akt (1:3000; Cell Signaling) or anti-β-actin (1:4000; Applied Biological Materials, Richmond, BC, Canada) antibody. Blots were detected using anti-rabbit HP-conjugated or anti-mouse HP secondary antibody (1:3000, Millipore, Billerica, MA, USA) and a chemiluminescence kit (Thermo Fisher Scientific, Waltham, MA, USA).

### Preparation of conditioned media

Cells were grown to subconfluency in gelatin-coated 12-well plates, starved for 12 h and then treated with OPNpt20 (1 μM) or OPNpt20-Db (1 μM) for 12 h with or without anti-α_v_β_3_ antibody (0.1 μg ml^−1^) or immunoglobulin G (IgG) (0.1 μg ml^−1^). Conditioned medium was harvested and centrifuged at 8000 r.p.m. for 2 min at room temperature to eliminate dead cells. Supernatants were then transferred to clean tubes, and proteins were measured by immunoblotting and gelatin zymography.

### Pull-down assay

Pull-down assays were performed using streptavidin agarose beads (Pierce, Rockford, IL, USA). Briefly, HUVEC lysates were preincubated with anti-α_v_β_3_ antibody (Abcam) or IgG (Santa Cruz Biotechnology) for 15 min at 4 °C with rotation and then incubated with biotinylated-OPNpt20 (bt-OPNpt20, 0.1 μg μl^−1^) or biotinylated-OPNpt20-RAA (bt-OPNpt20-RAA, 0.1 μg μl^−1^) for 30 min. These mixtures were incubated with 20 μl of streptavidin beads for 30 min at 4 °C, centrifuged at 8000 r.p.m. for 2 min, washed three times and analyzed by immunoblot using anti-integrin αV (1:1000; Santa Cruz Biotechnology) and anti-β-actin (1:4000; Applied Biological Materials) antibodies.

### Gelatin zymography

Conditioned medium from HUVEC cultures was adjusted to the same protein concentration and analyzed for gelatin digestion by electrophoresis under non-reducing conditions on SDS-polyacrylamide gels containing gelatin (1 mg ml^−1^). Gels were washed with washing buffer (50 mM Tris-HCl (pH 7.5), 2.5% Triton X-100, 5 mM CaCl_2_, 1 μM ZnCl_2_) to remove SDS from gels and, 30 min later, were incubated in incubation buffer (50 mM Tris-HCl (pH 7.5), 1% Triton X-100, 5 mM CaCl_2_, 1 μM ZnCl_2_) for 72 h at 37 °C. Gels were stained with staining solution (0.5% Coomassie brilliant blue, 40% methanol, 10% acetic acid) for 1 h at room temperature and then incubated with destaining solution (40% methanol, 10% acetic acid) until white degraded zones were clearly seen.

### Immunohistochemistry

Brains were fixed in 4% paraformaldehyde solution for 2 days at 4 °C and postfixed in 30% sucrose solution at 4 °C. Brain sections were obtained by sectioning at 30 μm using a vibratome, and immunological staining was performed. Sections were then blocked with 5% FBS, 5% horse serum and 2% albumin in 0.1% Triton X-100 for 1 h at room temperature. Primary antibodies anti-rat endothelial cell antigen-1 (RECA-1) (AbD Serotec, Kidlington, UK) and anti-α-smooth muscle actin (Santa Cruz Biotechnology) were used at a concentration of 1:500. After incubation with primary antibodies, brain sections were washed with PBS and incubated with rhodamine-labeled anti-rabbit IgG (1:300; Jackson ImmunoRes, West Grove, PA) secondary antibody. Sections were then mounted with mounting solution containing DAPI (Vector Laboratories, Peterborough, UK), and endothelial areas were analyzed using the AngioTool Software (National Cancer Institute, Gaithersburg, MD, USA).

### Tissue preparation and immunohistochemistry for hypoxyprobe adducts

To detect hypoxic areas in brain, rats were injected intraperitoneally with hypoxyprobeTM-1 (60 mg kg^−1^, solid pimonidazole hydrochloride; Natural Pharmacia International, Burlington, MA, USA) at 3 h before being killed. Isolated brains were immediately immersed in 4% paraformaldehyde for 2 days at 4 °C and postfixed in 30% sucrose solution at 4 °C. Brains were then sectioned and stained with fluorescein isothiocyanate -conjugated anti-pimonidazole antibody.

### Statistical analysis

Statistical analysis was performed using the two-tailed Student’s *t*-test. The results are presented as the mean±s.e.m., and statistical significance was accepted for *P*-values <0.05.

## Results

### OPNpt20 induced HUVEC proliferation, and its RGD and SLAY motifs had critical roles

Angiogenesis is a complex biological process that requires the precise coordination of proliferation, migration and tube formation by endothelial cells. To determine whether OPNpt20 (Figure 1a) induces endothelial cell proliferation, HUVECs were treated with 0.01, 0.1 or 1 μM of OPNpt20 for 24 h, and the total number of Ki67-positive cells were counted to measure cell proliferation. Ki67-positive cell numbers were found to increase in an OPNpt20 dose-dependent manner, and 1 μM of OPNpt20 induced a 4.8-fold increase versus treatment-naïve controls (Figure 1b and c). To examine the involvement of the RGD and SLAY motifs of OPNpt20 in HUVEC proliferation, cells were treated with 1 μM of three different mutant OPNpt20 peptides (Figure 1a), in which RGD was replaced with RAA (OPNpt20-RAA), SLAY was replaced with SLAA (OPNpt20-SLAA) or RGDSLAY was replaced with RAASLAA (OPNpt20-Db), for 24 h. HUVEC proliferation was reduced to 63.0±5.7% or 26.8±4.2% in OPNpt20-RAA- and OPNpt20-Db-treated cells, respectively, of that in OPNpt20-treated cells but not in OPNpt20-SLAA-treated cells (Figure 1e and f). OPNpt20-mediated HUVEC proliferation was confirmed by the MTT assay, and compared with OPNpt20-treated cells, the results showed that cell survival was lower for OPNpt20-RAA- and OPNpt20-Db-treated cells but not for OPNpt20-SLAA-treated cells (Figure 1d and g). These results indicate that OPNpt20 induced HUVEC proliferation, that the RGD motif of OPNpt20 has a critical role and that the SLAY motif also contributes to this process but to a lesser extent.

### OPNpt20 induced HUVEC migration in an RGD and SLAY motif-dependent manner

**Figure 2:**
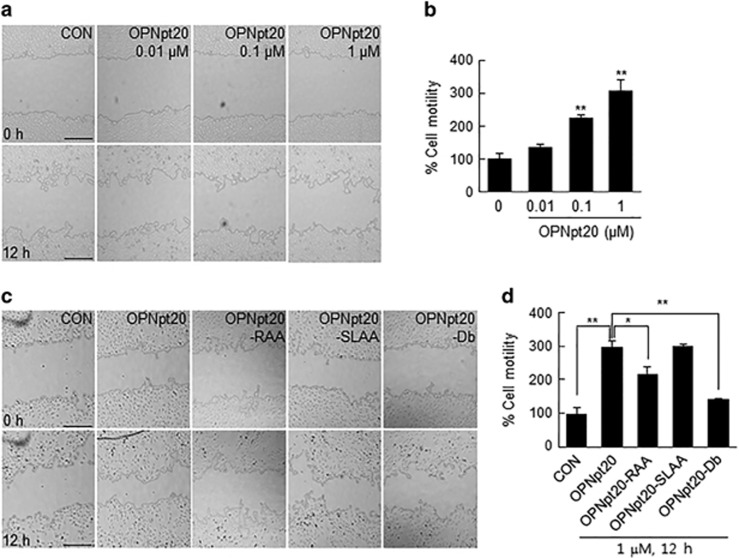
Induction of cell migration by 20-amino-acid OPN peptide (OPNpt20) and its mutants in human umbilical vein endothelial cell (HUVEC) cultures. (**a**, **b**) Cell migration was evaluated using a wound healing assay in HUVEC cultures after incubation with OPNpt20 (0.01, 0.1 or 1 μM) for 12 h. (**b**) Cell motility was assessed by measuring wound widths at 0 and 12 h. The results are presented as the mean±s.e.m. (*n*=4). (**c**, **d**) HUVEC migration was measured after treatment with the three mutant peptides (1 μM) for 12 h. Representative images are presented (**a**, **c**), and results are presented as the mean±s.e.m. (*n*=3) (**b**, **d**). Scale bars, 400 μm. *P<0.05, **P<0.01 between the indicated groups.

To determine whether OPNpt20 induces HUVEC migration, we used a wound healing assay after treating cells with OPNpt20 (0.01, 0.1 or 1 μM) or its three mutant peptides (1 μM) for 12 h. Cell motility was determined by measuring wound widths and was found to increase in a dose-dependent manner in OPNpt20-treated cells (Figure 2a and b). At 1 μM of OPNpt20, cell migration increased to 299.6±16.7% of that of treatment-naïve controls (Figure 2c and d). However, cell migration in OPNpt20-RAA- and OPNpt20-Db-treated cells was reduced to 72.9±6.4% and 47.0±1.2%, respectively, of that in OPNpt20-treated cells, while cell migration in OPNpt20-SLAA-treated cells was comparable to that in OPNpt20-treated cells (Figure 2c and d). These results indicate that OPNpt20 induced HUVEC migration, its RGD motif had a critical role and its SLAY motif had a lesser role.

### OPNpt20 induced tube formation by HUVECs in an endogenous α_v_β_3_-integrin-dependent manner

**Figure 3:**
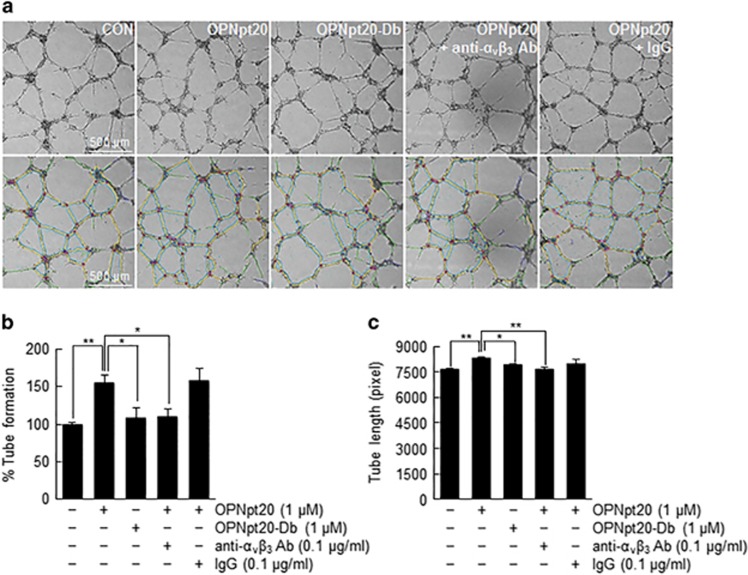
Induction of tube formation by 20-amino-acid OPN peptide (OPNpt20) in human umbilical vein endothelial cell (HUVEC) cultures. OPNpt20 (1 μM)-induced tube formation was examined in HUVECs treated with OPNpt20 (1 μM) or OPNpt20-Db (mutant OPN peptide with both RGD and SLAY replaced; 1 μM) for 12 h in the presence or absence of anti-α_v_β_3_ antibody or immunoglobulin G (IgG). Representative images obtained from Image J analyzer (National Institute of Health, Bethesda, MD, USA) are presented (green, branches; yellow, master segments; blue, tubes; red, master junctions) (**a**). Tube numbers were counted (**b**), and tube lengths were measured (**c**). The results are presented as the mean±s.e.m. (*n*=3). Scale bars, 500 μm. *P<0.05, **P<0.01 between the indicated groups.

To confirm the proangiogenic effects of OPNpt20, we examined tube formation by HUVECs. Tube-like structures harboring branches, segments and nodes were observed after culturing HUVECs on Matrigel for 12 h (Figure 3a). OPNpt20 (1 μM) increased tube formation to 155.3±9.4% versus treatment-naïve control cells, but treatment with OPNpt20-Db did not enhance tube formation (Figure 3a and b). Interestingly, tube formation did not increase when anti-α_v_β_3_-integrin antibody was cotreated with OPNpt20 (OPNpt20+anti-α_v_β_3_ antibody), but increased when IgG was cotreated (OPNpt20+IgG) (Figure 3a and b), indicating that α_v_β_3_-integrin is critical for OPNpt20-mediated tube formation. Similarly, measurements taken after culturing HUVECs on Matrigel for 12 h revealed that total tube lengths were also significantly increased in the presence of the RGDSLAY motif and α_v_β_3_-integrin, which confirmed the notion that endogenous α_v_β_3_-integrin is required for OPNpt20-mediated tube formation (Figure 3a and c).

### The interaction between OPNpt20 and endogenous α_v_β_3_-integrin mediated the proangiogenic effect of OPNpt20 in HUVECs

**Figure 4:**
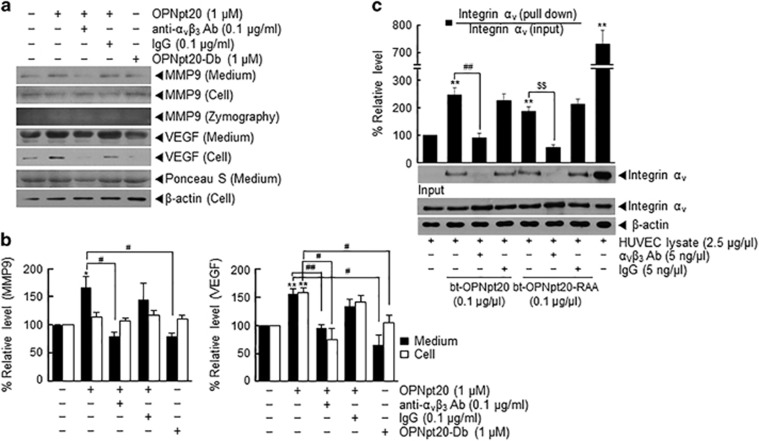
Endogenous α_v_β_3_-integrin mediates the proangiogenic effect of 20-amino-acid OPN peptide (OPNpt20) in human umbilical vein endothelial cells (HUVECs). (**a**, **b**) Protein levels of matrix metallopeptidase 9 (MMP9) and vascular endothelial growth factor (VEGF) in conditioned media or HUVEC lysates were assessed by immunoblotting after treatment with OPNpt20 (1 μM) or OPNpt20-Db (mutant OPN peptide with both RGD and SLAY replaced; 1 μM) for 12 h in the presence or absence of anti-α_v_β_3_ (0.1 μg ml^−1^) antibody or IgG (0.1 μg ml^−1^). The results are presented as the mean±s.e.m. (*n*=3) (**b**). (**c**) Direct binding between endogenous integrin α_v_ and biotinylated-OPNpt20 or biotinylated-OPNpt20-RAA was examined using a pull-down assay. HUVEC lysates (2.5 μg μl^−1^) were preincubated with anti-α_v_β_3_ or IgG (0.5 ng μl^−1^) for 15 min and then incubated with biotinylated-OPNpt20 or biotinylated-OPNpt20-RAA for 30 min. Complexes were pulled down with streptavidin beads, and α_v_-integrin was measured by immunoblot using anti-α_v_-integrin antibody. Representative images are presented and results are presented as the mean±s.e.m. (*n*=4) (**c**). *P<0.05, **P<0.01 versus treatment-naïve controls, ^#^*P*<0.05, ^##^*P*<0.01, ^$$^*P*<0.01 between the indicated groups.

Protein levels of MMP9 and VEGF (proangiogenic markers) in media were higher for OPNpt20-treated HUVECs than for treatment-naïve controls (Figure 4a and b). Conversely, accumulation of MMP9 and VEGF in OPNpt20-treated HUVEC medium was significantly reduced by cotreating cells with anti-α_v_β_3_ antibody but not by cotreating with IgG (Figure 4a and b), indicating that the interaction between OPNpt20 and α_v_β_3_-integrin also has an important role in the induction of MMP9 or VEGF in HUVECs. Induction of MMP9 was confirmed with zymography (Figure 4a). As expected, OPNpt20-Db failed to induce upregulation of MMP9 and VEGF (Figure 4a and b). Furthermore, when a pull-down assay was performed using bt-OPNpt20, an interaction was detected between bt-OPNpt20 and endogenous integrin α_v_ in HUVEC lysates. However, this interaction was not detected when HUVEC lysates was preincubated with anti-α_v_β_3_-integrin antibody (Figure 4c). This interaction with endogenous integrin α_v_ was also detected when bt-OPNpt20-RAA was used instead of bt-OPNpt20, but not after preincubating HUVEC lysates with anti-α_v_β_3_ integrin antibody (Figure 4c). These results show that exogenous OPNpt20 and endogenous α_v_β_3_ integrin interact, and this interaction might be responsible for the proangiogenic effect of OPNpt20 in HUVECs.

### α_v_β_3_-Integrin/AKT and ERK signaling pathways were involved in OPNpt20-mediated angiogenesis

**Figure 5:**
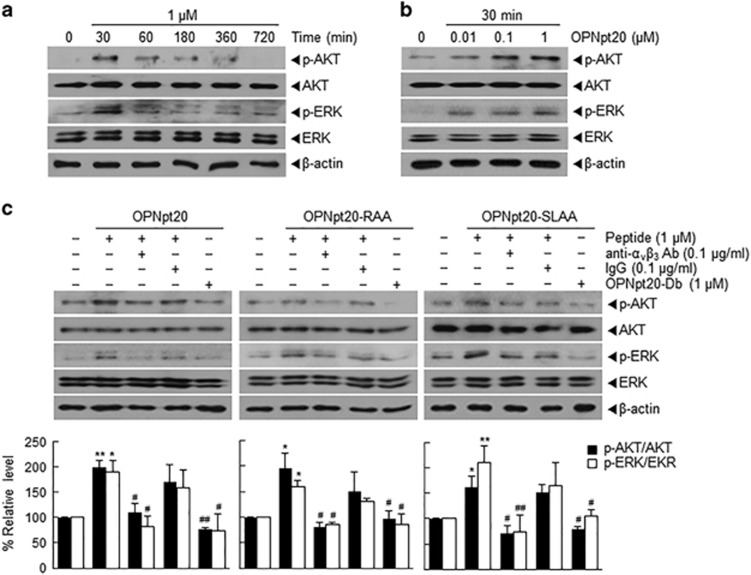
Activation of ERK and AKT pathways by 20-amino-acid OPN peptide (OPNpt20) in human umbilical vein endothelial cellS (HUVECs) and the role played by α_v_β_3_-integrin. (**a**, **b**) HUVECs were incubated with OPNpt20 (1 μM) for 30, 60, 180 or 360 min (**a**) or with 0.01, 0.1 or 1 μM of OPNpt20 for 1 h (**b**), and total and phosphorylated-ERK and -AKT levels were assessed by immunoblotting. (**c**) HUVECs were incubated with OPNpt20 (1 μM) or its three mutant peptides for 30 min in the presence or absence of anti-α_v_β_3_ antibody or IgG, and total and phosphorylated-ERK and -AKT levels were assessed by immunoblotting. Representative images are presented, and results are presented as the mean±s.e.m. (*n*=3). *P<0.05, **P<0.01 versus treatment-naïve controls, ^#^*P*<0.05, ^##^*P*<0.01 versus OPNpt20-, OPNpt20-RAA or OPNpt20-SLAA-treated cells.

PI3K, AKT and ERK signaling pathways are involved in OPN-induced angiogenesis in HUVEC cultures.^18^ We found that in OPNpt20-treated HUVEC cultures, ERK and AKT were phosphorylated within 30 min of treatment (Figure 5a), and maximum phosphorylation occurred at OPNpt20 concentrations of 0.1 or 1 μM, respectively (Figure 5b). Furthermore, phosphorylation of ERK and AKT was suppressed by pretreating cells with anti-α_v_β_3_ antibody (100 ng ml^−1^) for 30 min but not by pretreating cells with IgG (100 ng ml^−1^, 30 min) (Figure 5c). The phosphorylation of ERK and AKT was also observed in OPNpt20-RAA- or OPNpt20-SLAA-treated cells and suppressed by pretreating with anti-α_v_β_3_ antibody (Figure 5c). However, the phosphorylation of ERK and AKT were not detected in OPNpt20-Db-treated cells. Taken together, these results indicated that endogenous α_v_β_3_-integrin interacts with the RGD and SLAY motifs of OPNpt20 and that these interactions are important for activating AKT and ERK signaling pathways in HUVECs.

### Proangiogenic effects of OPNpt20 in the postischemic brain

**Figure 6:**
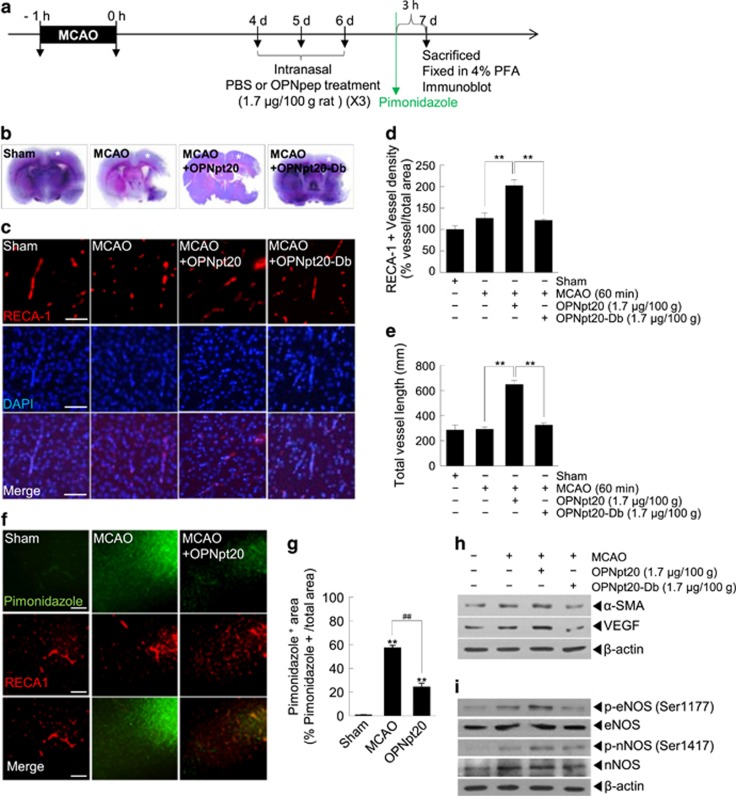
Induction of angiogenesis and of proangiogenic markers by 20-amino-acid OPN peptide (OPNpt20) in postischemic brains. (**a**) Schematic of the experiment. (**b**–**e**) OPNpt20 or OPNpt20-Db (mutant OPN peptide with both RGD and SLAY replaced; 1.7 μg/100 g body weight) were administered intranasally three times at 4, 5 and 6 days after 60 min of middle cerebral artery occlusion (MCAO). Seven days after MCAO, coronal brain sections were stained using cresyl violet (**b**) or double fluorescence-stained with anti-rat endothelial cell antigen-1 (RECA-1) antibody and DAPI (4',6-diamidino-2-phenylindole) (**c**). Asterisks in (**b**) indicated areas examined in double fluorescent staining. Representative images are presented (**c**), and RECA-1-positive vessel densities are presented as the mean±s.e.m. (*n*=5) (**d**). (**e**) Total vessel length was measured using the AngioTool Software (National Cancer Institute, Gaithersburg, MD, USA), and results are presented as the mean±s.e.m. (*n*=5). Scale bars, 250 μm. **P<0.01 between the indicated groups. (**f**, **g**) OPNpt20 (1.7 μg/100 g body weight) was administered intranasally three times at 4, 5 and 6 days after 60 min of MCAO and hypoxyprobe (pimonidazole, 60 mg kg^−1^) was injected (intraperitoneally (i.p.)) 3 h before killing at 7 days after MCAO (**a**). Coronal brain sections were prepared and immunostained with fluorescein isothiocyanate (FITC)-conjugated anti-pimonidazole antibody. Representative images are presented (**f**), and results are presented as the mean±s.e.m. (*n*=6) (**g**). (**h**, **i**) Tissue lysates were prepared from asterisked regions in (**b**) at 7 days after MCAO and immunoblotted for vascular endothelial growth factor (VEGF), α-smooth muscle actin (SMA), total or phosphorylated endothelial NOS (Enos), total or phosphorylated neuronal NOS (nNOS) and β-actin. The results are representative of three independent experiments.

We also examined whether OPNpt20 exerts proangiogenic effects in the postischemic brain using a rat MCAO model. To confirm that OPNpt20 has a direct proangiogenic effect rather than an indirect effect caused by its neuroprotective effects, OPNpt20 (1.7 μg/100 g body weight) was administered intranasally at 4, 5 and 6 days after MCAO, and vessel formation was examined at 7 days after MCAO (Figure 6a). Infarct volumes in the OPNpt20- and OPNpt20-Db-administered MCAO groups were similar to those in PBS-administered MCAO controls, as determined by cresyl violet staining at 7 days after MCAO (Figure 6b). Blood vessel densities in cortical penumbra of ipsilateral hemispheres (Figure 6b, asterisk) were measured by immunostaining using anti- RECA-1 (a marker of endothelial cells) antibody. RECA-1-positive vessel density was slightly higher in treatment-naïve MCAO controls than in sham controls at 7 days after MCAO, and these densities were significantly enhanced in the OPNpt20-administered MCAO animals (160.8±12.0% of that in treatment-naïve MCAO controls) (Figure 6c and d). However, OPNpt20-Db did not increase vessel density (Figure 6c and d). Similarly, total vessel length was markedly increased in the OPNpt20-administered MCAO animals (226.1±11.2% of treatment-naïve MCAO controls), but not in the OPNpt20-Db animal (Figure 6e). Taken together, these results demonstrate a robust proangiogenic effect of OPNpt20 in the postischemic brain. To determine whether OPNpt20 induces functional blood vessels, hypoxia levels in the cortical penumbra of the ipsilateral hemisphere (asterisk in Figure 6a) were measured using a hypoxyprobe (pimonidazole) (Figure 6a). We found that hypoxia was significantly lower in OPNpt20-administered MCAO animals, 42.7±8.1% of that in PBS-treated MCAO controls (Figure 6f and g), indicating that the new vessels functioned properly. In addition, levels of angiogenesis-associated proteins, such as VEGF and α-smooth muscle actin, were also found to be upregulated in the same brain areas in the OPNpt20-administered group at 7 days after MCAO, but not in the OPNpt20-Db-administered MCAO group (Figure 6h). Furthermore, eNOS phosphorylation (Ser1177), which was induced in the postischemic brain, was further enhanced by OPNpt20, and similar enhancement of phospho-nNOS (Ser 1417) induction was detected in OPNpt20-treated animals (Figure 6i). These results confirm the proangiogenic potency of OPNpt20 in the postischemic brain and the importance of the contributions made by RGD and SLAY.

## Discussion

In a previous study, we reported that intranasally delivered OPNpt20 had a robust neuroprotective effect in the postischemic brain because of an anti-inflammatory effect mediated by the interaction between the RGD motif of OPNpt20 and α_v_β_3_-integrin.^16^ In the present study, we found that OPNpt20 has *in vitro* and *in vivo* proangiogenic effects. Although the proangiogenic effect of OPN has been documented under various pathophysiological conditions, including in various cancers,^19, 20^ the results obtained in the present study show that the exogenous OPN icosamer peptide, OPNpt20, binds to endogenous α_v_β_3_-integrin and induces proangiogenic effects *in vitro* and in the postischemic brain using mutant peptides and blocking antibodies.

In the present study, we found that the RGD motif has a critical role and that the SLAY motif also contributes to a lesser degree to the proangiogenic effect of OPNpt20, which is in keeping with previous reports showing that the RGD motif of OPN is essential for OPN-induced angiogenesis. It has been reported that an N-terminal fragment of OPN containing its RGD motif promoted adhesion of mouse and human fibroblasts more effectively than full-length OPN.^21^ In addition, cell attachment and spreading stimulated by an N-terminal fragment of OPN was inhibited by soluble GRGDS peptides or an OPN-specific antibody raised against the GRGDS domain of OPN.^22^ Furthermore, recent reports have shown that OPN and integrin has essential roles in angiogenesis in many tumor types.^19, 20^ In the present study, we showed that the RGD motif of OPNpt20 interacts with endogenous α_v_β_3_-integrin and that this interaction is important for activating AKT and ERK signaling pathways and subsequent angiogenic processes, such as tube formation, in HUVECs.

In terms of the SLAY motif of OPN, the proangiogenic activity of the SVVYGLR peptide has been reported in transformed rat lung endothelial cells,^23^ in a dorsal air sac model,^24^ in animal models of heart failure^25^ and in myocardial fibrosis.^26^ Furthermore, the SLAY motif has been reported to bind to various integrins, such as α_v_β_3_, α_4_β_1_, α_4_β_7_ or α_9_β_1,_ in a cell-type-dependent manner but HUVEC.^11, 12, 27, 28^ As the SLAY motif is located adjacent to the RGD sequence, the RGD and SLAY motifs in OPNpt20 appear to compete for binding with α_v_β_3_-integrin. However, in the present study, OPNpt20-RAA was found to have greater angiogenic potency than OPNpt20-Db, but comparable potency was observed for OPNpt20-SLAA and OPNpt20 (Figures 1 and 2). Based on these observations, we speculate that the RGD motif binds preferentially with α_v_β_3_-integrin and thus has a critical role, but in the absence of the RGD motif, the SLAY motif has a proangiogenic role. Furthermore, the possibility that integrins other than α_v_β_3_ bind to these two motifs cannot be excluded, and this possibility needs to be further explored.

In the present study, OPNpt20 conferred proangiogenic effects by activating the ERK and AKT signaling pathways (Figure 5), which is in accordance with a previous report regarding the involvement of ERK and AKT in OPN-mediated angiogenesis.^18^ Significant reductions in phosphorylated-ERK and phosphorylated-AKT levels after treatment with α_v_β_3_-integrin antibody in OPNpt20-, OPNpt20-RAA- and OPNpt20-SLAA-treated cells (Figure 5c) indicated that these signaling pathways are used in both RGD- and SLAY-mediated angiogenic processes. Furthermore, since a positive feedback loop has been reported between OPN-mediated VEGF induction and VEGF-mediated OPN induction, in which the PI3K/AKT and ERK pathways has important roles,^18^ it is possible that OPNpt20 might be able to trigger these positive feedback signals. In addition, since the phosphorylation of Ser 1177 or Ser 1417 of eNOS and nNOS, respectively, have been suggested to attenuate ischemic injury,^29, 30^ marked induction and activation of eNOS and nNOS by OPNpt20 might underlie its proangiogenic effects.

In the postischemic brain, angiogenesis at 7 days after MCAO was significantly induced when OPNpt20 was administered at 4, 5 and 6 days after MCAO (Figure 6), which was probably too late to affect infarct volumes. This suggested that OPNpt20 induced angiogenesis independent of its neuroprotective effects. Interestingly, OPN is induced in a delayed manner in various animal models of stroke. For example, in a mouse model of permanent focal ischemia, OPN induction began at 12 h and peaked at 5 days, and in a rat model of transient forebrain ischemia, induction began at 6 h and peaked at 5 days.^8^ In addition, delayed induction of α_v_β_3_-integrin in peri-infarct astrocytes after 5 and 15 days has been reported in a postpermanent rat model of MCA,^31^ which suggests that OPN–integrin interactions have important functions during the later period in the postischemic brain. This raises the possibility that administration of exogenous OPNpt20, which has effects comparable to OPN at earlier times, could accelerate repair processes in the postischemic brain. Furthermore, OPNpt20-mediated VEGF induction (Figures 4 and 6 and Supplementary Figure 1) might initiate a second round of proangiogenic processes. In terms of the efficiency of OPNpt20 delivery into the brain, we detected exogenously delivered fluorescein isothiocyanate-conjugated OPNpt20 in cerebral cortices 2 and 8 h after administration and its colocalization with endothelial cells and α_v_-integrin (Supplementary Figure 2). Further studies are required to identify specific interactions with different cell types, brain regions and times.

Because OPN is a multifunctional protein, it is possible that OPNpt20 might have effects other than anti-inflammatory^16^ and proangiogenic effects, such as antiapoptotic effects,^32^ the scavenging of toxic substances like excess Ca^2+^ or dead neurons,^33, 34^ or attractant/inducer functions that drive the migration of neuroblasts from the subventricular zone to ischemic regions.^35^ Although additional studies are required to determine whether OPNpt20 indeed performs the above-mentioned functions, it offers great promise as a multifunctional therapeutic agent. Compared with whole OPN, the greater diffusion of OPNpt20 and its enhanced interaction with α_v_β_3_-integrin, which is caused by mobilization of its C terminus, increase its proangiogenic potency. Furthermore, OPNpt20 is derived from an endogenous protein, reducing safety concerns.

## Publisher’s note

Springer Nature remains neutral with regard to jurisdictional claims in published maps and institutional affiliations.
