# Supplementary material for: Proangiogenic functions of an RGD-SLAY-containing osteopontin icosamer peptide in HUVECs and in the postischemic brain
Source: Exp Mol Med. 2018 Jan 19;50(1):e430–. doi: 10.1038/emm.2017.241 (PMC5799800; doi:10.1038/emm.2017.241)
Supplement: Supplementary Information [file emm2017241x1.pdf]

Supplementary information

Proangiogenic functions of RGD-SLAY-containing osteopontin icosamer peptide in HUVECs and in the postischemic brain

Hahnbie Lee[a,b], Yin-Chuan Jin[a,c], Seung-Woo Kim[a,b], Il-Doo Kim[a,b], Hye-Kyung Lee[a,b], Ja-Kyeong Lee[a,b]

[a]Department of Anatomy, [b]Medical Research Center, Inha University School of Medicine, Inchon, Korea; [c]Department of Histology and Embryology, Binzhou Medical University, Yantai 264000, China

*Corresponding author:

Ja-Kyeong Lee, Ph.D.

Department of Anatomy,

Inha University School of Medicine,

Inharo 100 Nam-gu

Inchon, 22212, Republic of Korea

Tel: +82-32-890-0913

FAX: +82-32-884-2105

jklee@inha.ac.kr

Supplementary figure 1. Accumulation of VEGF and MMP9 in sera and cerebrospinal fluids of OPNpt20-treated animals after MCAO

Levels of VEGF and MMP9 were examined in sera (a) or in cerebrospinal fluid (CSF) (b) obtained from sham control, treatment-naïve MCAO control, OPNpt20-administered MACO group, or OPNpt20-Db-administered MACO group by immunoblotting at 7 days post-MCAO.

Supplementary figure 2. Detection of intranasally administered OPNpt20 in the postischemic brain

(a) PBS or FITC-labeled OPNpt20 (1.7 μg/100 g) was administered intranasally at 4 days post-MCAO and brain sections (b) were immunostained with anti-RECA-1 and anti-α_v integrin antibodies at 2 or 8 h after PBS (c) or OPNpt20 (d-e) administration. Localizations of FITC signal and RECA-1- and/or α_v integrin-positive cells in cortical penumbra (asterisk in b) were visualized using fluorescent microscopy. DAPI staining was conducted to visualize nuclei. Arrow heads indicate FITC signal and arrows indicate merged signal for FITC signal, RECA-1 immunoreactivity, and α_v integrin immunoreactivity. Scale bars present 20 μm.
