# Supplementary figures and images for: Proangiogenic functions of an RGD-SLAY-containing osteopontin icosamer peptide in HUVECs and in the postischemic brain
Source: Exp Mol Med. 2018 Jan 19;50(1):e430–. doi: 10.1038/emm.2017.241 (PMC5799800; doi:10.1038/emm.2017.241)

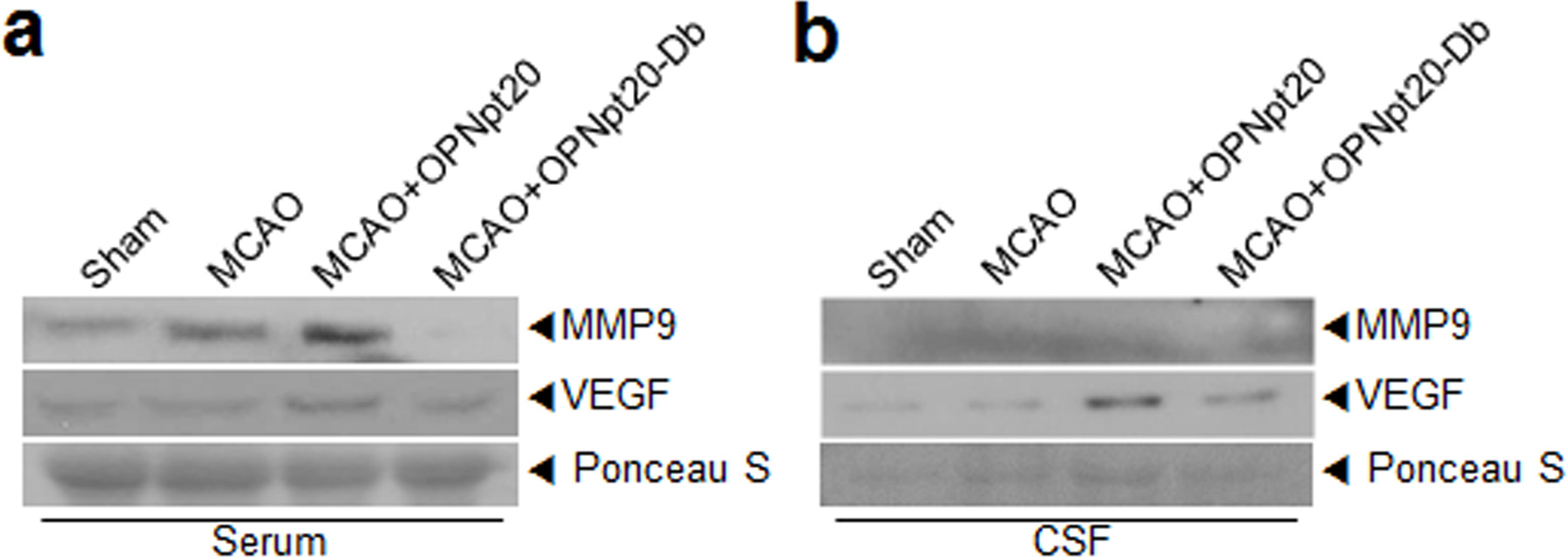

Supplement: Supplementary Figure 1 [file emm2017241x2.tif]

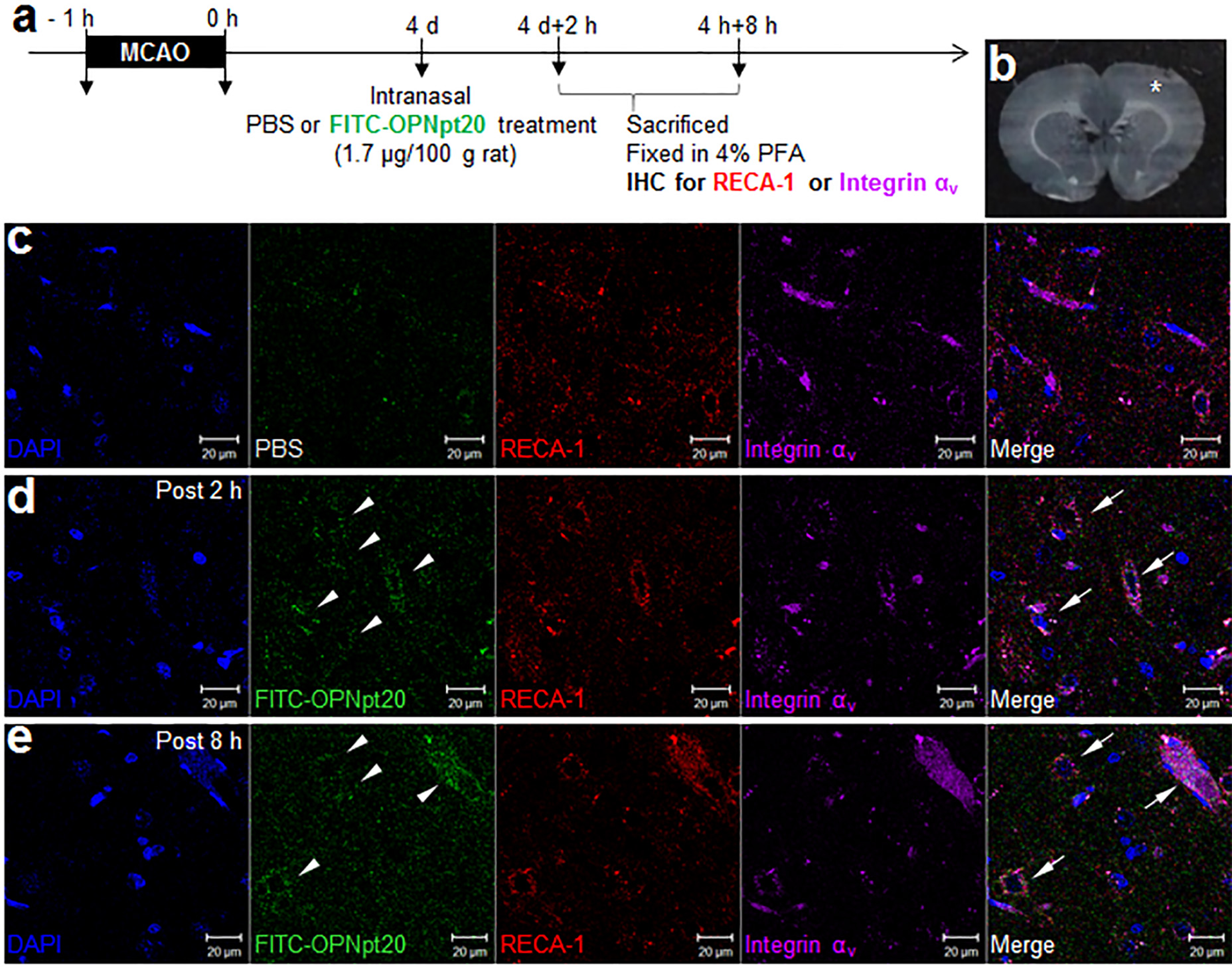

Supplement: Supplementary Figure 2 [file emm2017241x3.tif]
